# Seroprevalence and Risk Factors of *Chlamydia abortus* Infection in Tibetan Sheep in Gansu Province, Northwest China

**DOI:** 10.1155/2014/193464

**Published:** 2014-10-23

**Authors:** Si-Yuan Qin, Ming-Yang Yin, Wei Cong, Dong-Hui Zhou, Xiao-Xuan Zhang, Quan Zhao, Xing-Quan Zhu, Ji-Zhang Zhou, Ai-Dong Qian

**Affiliations:** ^1^College of Animal Science and Technology, Jilin Agricultural University, Changchun, Jilin Province 130118, China; ^2^State Key Laboratory of Veterinary Etiological Biology, Lanzhou Veterinary Research Institute, Chinese Academy of Agricultural Sciences, Lanzhou, Gansu Province 730046, China

## Abstract

*Chlamydia abortus*, an important pathogen in a variety of animals, is associated with abortion in sheep. In the present study, 1732 blood samples, collected from Tibetan sheep between June 2013 and April 2014, were examined by the indirect hemagglutination (IHA) test, aiming to evaluate the seroprevalence and risk factors of *C. abortus* infection in Tibetan sheep. 323 of 1732 (18.65%) samples were seropositive for *C. abortus* antibodies at the cut-off of 1 : 16. A multivariate logistic regression analysis was used to evaluate the risk factors associated with seroprevalence, which could provide foundation to prevent and control *C. abortus* infection in Tibetan sheep. Gender of Tibetan sheep was left out of the final model because it is not significant in the logistic regression analysis (*P* > 0.05). Region, season, and age were considered as major risk factors associated with *C. abortus* infection in Tibetan sheep. Our study revealed a widespread and high prevalence of *C. abortus* infection in Tibetan sheep in Gansu province, northwest China, with higher exposure risk in different seasons and ages and distinct geographical distribution.

## 1. Introduction

*Chlamydia*, an obligate intracellular gram-negative bacterium, is known to cause a variety of diseases in animals and humans [1, 2]. Chlamydiaceae have a single genus* Chlamydia* that includes nine species; among them* C. abortus* and* C. pecorum* can cause diseases in sheep [1–3]. In particular,* C. abortus* is recognized as a major cause of abortion and lamb loss throughout the world, especially in the intensively managed farms [4–6].* C. abortus* usually causes ulceration of endometrial epithelium resulting in placental infection if infection was acquired during the early stages of that pregnancy. More typically, infection acquired during late gestation will result in abortion in the following gestation and the symptoms caused by* C. abortus* also include epididymitis, pneumonia, arthritis, and conjunctivitis [7–9]. Recent reports described the presence of* C. abortus* DNA in the eyes of ewes [10, 11].* C. abortus* not only causes economic loss in the sheep industry, but also induces abortions in humans due to contact with aborting sheep or goats [12–14].

Chlamydial infection of sheep has been reported throughout the world [10, 15–18], including China. However, on account of Tibetan sheep breeding only in northwest China, no more reports were available on epidemiology of* C. abortus* infection in Tibetan sheep in China except that several reports about* C. abortus* infection in Tibetan sheep in Qinghai were published in local journals [19, 20] and only one article concerning* C. abortus* infection in Tibetan sheep in Tibet was documented in the international literature [21].

Tibetan sheep is one of the three main original sheep breeds living in the Qinghai-Tibetan Plateau passing through parts of regions of Gansu province, northwest China, where the solar radiation is strong and the temperature and oxygen content are low [19]. Tibetan sheep are recognized as a major source of income for local Tibetans, due to its high quality pelage and nutritive and delicious meat. However, it is yet to know whether Tibetan sheep are infected with* C. abortus *in Gansu Province, northwestern China. The objective of the present study was to evaluate the seroprevalence and risk factors of* C. abortus* infection in Tibetan sheep in Tianzhu Tibetan Autonomous County and Gannan Tibetan Autonomous Prefecture, Gansu province, northwest China.

## 2. Materials and Methods

### 2.1. The Study Site

The serum samples used in the present study were collected from Tibetan sheep in Tianzhu Tibetan Autonomous County and Gannan Tibetan Autonomous Prefecture including Maqu County and Luqu County in Gansu Province, northwest China. The Tibetan sheep in Gansu were mainly bred in Tianzhu and Gannan, in which most of the Tibetans also live. The sampling site is between the eastern longitudes of 102°07′-103°46′ and northern latitudes of 36°31′–37°55′ in Tianzhu Tibetan Autonomous County, and the average annual temperatures are from −8 to 4°C. Gannan Tibetan Autonomous Prefecture is located between longitudes 100°45′ to 104°45′ east and latitudes 33°06′ to 35°34′ north, in which the average annual temperatures are from 1 to 13°C.

### 2.2. Serum Samples

Blood samples were collected from 1732 Tibetan sheep in 3 counties in Gansu province from June 2013 to April 2014. The samples were collected randomly from 8 Tibetan sheep herds including 4 random herds in Tianzhu Tibetan Autonomous County and 4 random herds of the farms in Gannan Tibetan Autonomous Prefecture. Serum samples were separated from blood samples in local veterinary stations and then were taken to the laboratory and stored at −20°C until further tested. The detailed information of animals was obtained from local herdsmen by questioning.

### 2.3. Serological Examination

A commercially available Indirect Hemagglutination Assay (IHA) kit (Lanzhou Veterinary Research Institute, Chinese Academy of Agricultural Sciences, Lanzhou, China) was purchased to test antibodies to* C. abortus* and it was carried out according to the manufacturer's instructions as described previously [20–22]. The IHA kit was employed following the standard method described by China CADC, and the sensitivity and specificity of the experiment are 100% and 95%, respectively. The Ministry of Agriculture of China (NY/T 562-2002) has validated the sensitivity and specificity values for the testing kit used in this study. In brief, serum samples were added to 96-well V-bottomed polystyrene plates, which were diluted 4-fold serially beginning with 1 : 4 to 1 : 1,024. Then, the* C. abortus* antigen was added, and the plates were shaken gently for 2 min and incubated at 37°C for 2 h. Each test was performed with positive, negative, and blank controls, and serum samples which had positive reaction at dilutions of 1 : 16 or higher dilutions were considered positive for* C. abortus* antibodies. Positive results between 1 : 4 and 1 : 16 were considered “suspect” and were retested.

### 2.4. Statistical Analysis

Differences in the seroprevalence of* C. abortus* among Tibetan sheep of different geographical origins, genders, seasons, and age groups were analyzed with Chi-square tests using the SPSS software (SPSS Inc., IBM Corporation, Version 19, USA). The differences were considered statistically significant if *P* < 0.05 and the 95% confidence intervals (CI) are also calculated.

## 3. Results 

In the present study, 323 (18.65%) out of 1732 serum samples from Tibetan sheep in Gansu province were seropositive for* C. abortus* infection by IHA at a 1 : 16 cut-off (Table 1). As shown in Table 1, the seroprevalence of* C. abortus* infection in Tianzhu County (*n* = 962), Maqu County (*n* = 588), and Luqu County (*n* = 182) was 16.32%, 22.62%, and 18.13%, respectively. The numbers of seropositive animals and seroprevalence of individual herd were described in Table 2. All of the sampled 8 Tibetan sheep herds were serologically positive. Male sheep had a higher prevalence (21.51%) compared to females (17.39%). The positive samples were distributed among four seasons (Spring: *n* = 480; Summer: *n* = 398; Autumn: *n* = 479; Winter: *n* = 375) in which the prevalences of* C. abortus* infection were 18.54%, 22.11%, 20.46%, and 12.80% (*P* < 0.05, Table 1), respectively. The ages of the examined Tibetan sheep varied from 0 years to 3 years or greater and seroprevalence in different age groups ranged from 7.46% to 24.41% (Table 1). The highest seroprevalence was found in Tibetan sheep older than 3 years (24.41%) and the lowest in 0 years to 3 years (7.46%).  Table 3 summarizes the antibodies titers in different ages, regions, genders, and seasons with IHA titers of 1 : 16 in 188 samples (58.20%), 1 : 32 in 72 samples (22.29%), 1 : 64 in 34 samples (10.53%), 1 : 128 in 14 samples (4.33%), 1 : 256 in 12 samples (3.72%), 1 : 512 in 2 samples (0.62%), and 1 : 1024 in 1 sample (0.31%).

According to forward stepwise logistic regression, gender of Tibetan sheep was not significant in the logistic regression analysis (*P* > 0.05) and left out of the final model. Multivariable analysis of such 4 factors showed that* C. abortus* seropositivity was associated with regions, seasons, and ages. Tibetan sheep in Luqu County was more than 1.4-fold increase (OR = 1.40, 95% CI = 1.07–1.84, *P* = 0.014) at risk of* C. abortus* infection compared to Tibetan sheep in Tianzhu County, and no regional differences were found in Luqu County compared to Tianzhu County (*P* > 0.05). Ages of Tibetan sheep which were 1 < years ≤ 2 years old (15.82%) had a 2.5-fold increase higher risk of being seropositive compared to that of Tibetan sheep which were 0 < years ≤ 1 years old (7.46%) (OR = 2.48, 95% CI = 1.51–4.07, *P* < 0.001), while Tibetan sheep of 2 < years ≤ 3 and >3 years old had a 3-fold increase (OR = 1.40, 95% CI = 1.72–5.19, *P* < 0.001) and 4.0 times (OR = 4.02, 95% CI = 2.58–6.26, *P* < 0.001) higher risk compared to Tibetan sheep of 0 < years ≤ 1 age group separately (Table 4). In terms of seasons, the risk of* C. abortus* infection in summer was more than 2.0-fold increase (OR = 1.97, 95% CI = 1.33–2.91, *P* = 0.001) compared to* C. abortus* infection in winter (Table 4).

## 4. Discussions

Members of the family Chlamydiaceae can result in a broad range of diseases in both humans and animals, including sheep, which could cause health problems and heavy economic losses [23]. In particular, lamb loss in sheep was caused by* C. abortus* in North America, Africa, and parts of Europe, including UK, in which costs to the farming industry due to* C. abortus* in sheep were estimated to be £ 20,000,000 per annum [1]. Tibetan sheep surveyed in the present study were free-ranging on the grassland in Tianzhu Tibetan Autonomous County and Gannan Tibetan Autonomous Prefecture, and the meat production of which was not only transported to other Cities of Gansu province including Lanzhou as the provincial capital, but also transported to adjacent provinces including Qinghai, Sichuan, Shanxi, and Ningxia. However, there was little information available about the prevalence of* C. abortus* in Tibetan sheep in Gansu province, northwest China.

In the present study, the overall* C. abortus* seroprevalence in Tibetan sheep in Gansu province was 18.65%, which is higher than the 12.69% seroprevalence among Tibetan sheep in Tianjun County [19] and 8.01% in Yushu County of Qinghai province, China, by IHA [24]. It is lower than the values of 20.9% in Tibetan sheep in Tibet [21] and 26.3% in sheep in Gansu, China [25]. The different prevalence observed was probably due to differences in animal-welfare, sanitation, climates, and husbandry practices. In view of herds surveyed, although seropositive samples were found in all examined herds, seroprevalence in each herd varied obviously. Many seropositive animals in one tested herd could have an effect on high level of seroprevalence in evaluated region and the high seroprevalence may not be connected with individual region but with high level of infection at the herd level, which may be the main season for high seroprevalence in Gannan.

*Chlamydia* infection in humans and animals was caused by direct contacts with infected animals. The transmission of* Chlamydia* occurs mainly through inhalation or ingestion of these infectious dust contaminated materials [26]. Most of the time Tibetan sheep are free-ranging on the grassland and sheds of sheep are simple and crude, which increase the opportunity to be infected with* C. abortus*. The farms of Tibetan sheep herds were located in mountainous areas belonging to Qinghai-Tibet Plateau in which a lot of yaks lived around with Tibetan sheep. Yaks infected with* C. abortus *have been reported in Qinghai [22] and Gansu provinces (unpublished), which indicated a high risk as a source of* C. abortus* infection for Tibetan sheep. This may be one of the possible reasons for the high* C. abortus* seroprevalence (18.65%) in Tibetan sheep in Gansu, northwestern China.

In the present study, the* C. abortus* seroprevalence in male and female sheep was 21.51% and 17.39%, respectively. However, there was no significant difference in* C. abortus* seroprevalence between genders (*P* > 0.05), which is consistent with the studies of Huang et al. [21] in which they reported negative association between sex and* C. abortus* prevalence in Tibetan sheep in Tibet, implying that gender may not be a crucial factor for* C. abortus* infection in Tibetan sheep.

This study revealed that the geographical origin of Tibetan sheep is one of the risk factors associated with* C. abortus* seroprevalence. Tibetan sheep in Gannan Tibetan Autonomous Prefecture (21.56%) had a higher risk of being* C. abortus* seropositive compared to Tibetan sheep in Tianzhu Tibetan Autonomous County (16.32%). The geographic differences in prevalence may be related with differences in living environment and husbandry practices. According to local herders' introduction, wild animals including wild yaks, sika deer, and Tibetan antelope were found in field and farms of Gannan Tibetan Autonomous Prefecture, and all of these species are susceptible to* C. abortus*. In Gannan, people have low awareness of disease prevention and control, and it is difficult for veterinarians to reach the places to implement immunization programs. Therefore, living environment and husbandry practices were two main risk factors associated with* C. abortus*.

Season is a significant risk factor for* C. abortus* prevalence due to different climates in different seasons, including diverse temperature, precipitation, and humidity. In addition, the seasonal seroprevalence data may be related to persistence of antibody following abortion. Abortion occurred in spring resulting in generating many antibodies for resistance to* C. abortus* and the rubbish of abortion may be contacted by other healthy sheep, which led to high seroprevalence in summer. As time went on, the antibody level reached the lowest in winter.

Age of Tibetan sheep (years) as a continuous variable was analyzed in the logistic regression model, and the results showed that the prevalences were different significantly with ages, demonstrating that age is a predisposing factor for* C. abortus* prevalence. As the growth of the age, the seroprevalence of* C. abortus* infection went up all the time, indicating that there may be a cumulative likelihood for exposure to* C. abortus* infection with age in these surveyed regions.

IHA is regarded as a simple, safe, and useful method for examination of* C. abortus* antibodies, which has been employed in previous serological surveys [21, 22]. The sensitivity and specificity values of the testing IHA kit have been validated by the Ministry of Agriculture of China, which demonstrates that the IHA is not only more efficient than the CFT but also more inexpensive than the ELISA [27]. Due to these advantages, it may be the most appropriate commercially available kit for detecting* C. abortus* infection in Tibetan sheep.

## 5. Conclusions

Results of the present study revealed a high* C. abortus* seroprevalence in Tibetan sheep in Gansu province, which can cause significant economic losses to the local ovine industry and pose a potential threat to Tibetans in these areas. This study also showed that region, season, and age are main risk factors for* C. abortus* seroprevalence. Therefore, it is necessary to implement integrated control and efficient management measures to prevent and control* C. abortus* infection in Tibetan sheep in Gansu province.

## Figures and Tables

**Table 1:** Seroprevalence of *Chlamydia abortus* infection in Tibetan sheep associated with different factors in Gansu province, northwest China, by indirect hemagglutination assay (IHA).

Factor	Category	Tested numbers	Positive numbers	Prevalence (%)
Region	Tianzhu County	962	157	16.32
	Maqu County	588	133	22.62
	Luqu County	182	33	18.13
Sex	Male	530	114	21.51
	Female	1202	209	17.39
Season	Spring	480	89	18.54
	Summer	398	88	22.11
	Autumn	479	98	20.46
	Winter	375	48	12.80
Age (yr)	0 < yr ≤ 1	335	25	7.46
	1 < yr ≤ 2	392	62	15.82
	2 < yr ≤ 3	194	38	19.59
	yr > 3	811	198	24.41
Total		**1732**	**323**	**18.65**

**Table 2:** Seroprevalence of *Chlamydia  abortus* infection in Tibetan sheep in eight herds in two regions in Gansu, China.

Region	Herd numbers	Examined numbers	Positive numbers	Prevalence (%)
Tianzhu	Herd (1)	242	43	17.77
	Herd (2)	216	38	17.60
	Herd (3)	255	31	12.16
	Herd (4)	249	45	18.07
	Total	**962**	**157**	**16.32**
Gannan	Herd (5)	238	46	19.33
	Herd (6)	159	10	6.29
	Herd (7)	224	67	29.91
	Herd (8)	149	43	28.86
	Total	**770**	**166**	**21.56**

**Table 3:** Seroprevalence of *Chlamydia  abortus* infection in Tibetan sheep in Gansu, China, determined by indirect haemagglutination (IHA) test.

Biometric data	Category	Antibody titers							Positive numbers	Tested numbers	Prevalence (%)
		1 : 16	1 : 32	1 : 64	1 : 128	1 : 256	1 : 512	1 : 1024			
Age (years)	≤1	16	4	0	3	2	0	0	25	335	7.46
	1 < yr ≤ 2	39	12	7	2	2	0	0	62	392	15.82
	2 < yr ≤ 3	19	10	4	2	3	0	0	38	194	19.59
	yr > 3	114	46	23	7	5	2	1	198	811	24.41
Region	Tianzhu County	83	30	21	12	10	0	1	157	962	16.32
	Maqu County	85	35	8	2	1	2	0	133	588	22.62
	Luqu County	20	7	5	0	1	0	0	33	182	18.13
Sex	Male	76	24	9	2	2	1	0	114	530	21.51
	Female	112	48	25	12	10	1	1	209	1202	17.39
Season	Winter	28	10	4	3	2	0	1	48	375	12.80
	Spring	48	20	11	4	6	0	0	89	480	18.54
	Autumn	62	22	8	5	1	0	0	98	479	20.46
	Summer	50	20	11	2	3	2	0	88	398	22.11
	Total	**188**	**72**	**34**	**14**	**12**	**2**	**1**	**323**	**1732**	**18.65**

**Table 4:** Odds ratios for ages, seasons, and geographical origin of Tibetan sheep are taken as risk factors for *Chlamydia abortus* seroprevalence in Tibetan sheep.

Factor	Category	Tested numbers	Positive numbers	Prevalence (%)	OR (95% CI)	*P* value
Region	Tianzhu County	962	157	16.32	Reference	
	Maqu County	588	133	22.62	0.91 (0.56–1.48)	0.694
	Luqu County	182	33	18.13	1.40 (1.07–1.84)	0.014
Season	Winter	375	48	12.80	Reference	
	Spring	480	89	18.54	1.67 (1.08–2.57)	0.022
	Autumn	479	98	20.46	1.78 (1.21–2.62)	0.003
	Summer	398	88	22.11	1.97 (1.33–2.91)	0.001
Age (yr)	0 < yr ≤ 1	335	25	7.46	Reference	
	1 < yr ≤ 2	392	62	15.82	2.48 (1.51–4.07)	<0.001
	2 < yr ≤ 3	194	38	19.59	2.99 (1.72–5.19)	<0.001
	yr > 3	811	198	24.41	4.02 (2.58–6.26)	<0.001
